# SPRC19: A Database of State Policy Responses to COVID-19 in the United States

**DOI:** 10.1038/s41597-023-02343-4

**Published:** 2023-08-07

**Authors:** Frederick J. Boehmke, Bruce A. Desmarais, Abbie Eastman, Isabelle Grassel, Jeffrey J. Harden, Samuel Harper, Liam Kaboli, Hyein Ko, Elisabeth Oster, Tracee M. Saunders

**Affiliations:** 1 University of Iowa, Iowa City, IA 52242 USA; 2 Pennsylvania State University, State College, PA 16801 USA; 3 University of Notre Dame, Notre Dame, IN 46556 USA

**Keywords:** Government, Politics

## Abstract

SPRC19 is a new database that seeks to capture a wide range of state policy actions in response to COVID-19 in the United States. Since March 2020 we have monitored state governments’ and multi-state associations’ websites for executive orders, agency rules, new legislation, and court decisions. We categorize each policy action into one of 206 distinct policies, then document the branch of government, source document, announcement date, implementation date, and expiration date (if applicable). We also record whether the action represents the introduction of a new policy or the expansion or contraction of an existing policy. The current release of SPRC19, v3.0, captures over 13,000 distinct policy actions through April 2020, which constitutes thousands more actions than similar resources over the same time period.

## Background & Summary

Since the beginning of the COVID-19 pandemic, over six million total deaths have been confirmed. Of these, over one million have occurred in the Unites States. However, the rates of deaths and cases per state vary greatly. While the national average as of March 30, 2022 was just under three thousand cases per million, estimates across the states ranged from about one thousand in Hawaii to over four thousand in Mississippi. This variation reflects many differences across the states, including heterogeneity in demographics, baseline health, and geography. Because the U.S. policy response to COVID-19 has largely been left to the states, differences in deaths and cases, among others, also reflect differences in the policy choices made state governors, agencies, legislators, and courts. The policy actions taken by this variety of government officials reflect pandemic and political differences across the states, with the latter arising from differences in political ideology and state government structures. Understanding how state politics and institutions affect policy responses is critical for assessing the toll of COVID-19 and preparing for future pandemics and other emergencies.

State Policy Responses to COVID-19 (SPRC19) is a new database that seeks to provide a comprehensive record of all policy actions taken in the American states in response to COVID-19. We immediately began collecting these data at the outset of the pandemic in the spring of 2020. The data attempts to capture all state actions related to the pandemic and its consequences, including those that aim to directly reduce spread of the virus and those that address the consequences of those mitigation efforts. The policy choices made by state officials, including legislators, governors, and judges, occurred in real time in a fluid and dynamic environment. SPRC19 reflects this temporal variation by documenting policy *actions* rather than just the presence or absence of a given intervention. Similar to existing COVID-19 data collections, our data indicate when a state first adopted a particular policy and when it repealed or reinstated it. But unlike many of these SPRC19 also captures additional government actions: policy extension, expansion, reduction, and repeal. Our data reflect a deep interest in the political side of these decisions in addition to how they might affect public health or other individual outcomes. We believe this resource will also help researchers capture the broader, cumulative effects of state responses on public health outcomes.

SPRC19 is far from the first collection of Nonpharmaceutical Interventions (NPIs) available for the American states. Prominent examples of such collections include HIT-COVID^1^; the COVID-19 Control Strategies List (CCCSL)^2^; the COVID-19 Government Response Event Dataset (CoronaNet)^3^; the COVID-19 US State Policy Database (CUSP)^4,5^; the Oxford COVID-19 Government Response Tracker (OxCGRT)^6^; the COVID Analysis and Mapping of Policies (COVID AMP)^7^; and State-level social distancing policies in response to COVID-19 in the US^8^. As with other such collections such as these, SPRC19 has its relative advantages. Two such advantage arise from its broad coverage and its focus on the political process. In terms of coverage, SPRC19 includes over thirteen thousand actions for just the period January to April 2020, which is more actions than most other resources include in total through 2021 or 2022. It also includes 206 policy areas, which puts it at or near the top among similar resources. This large number of items reflects our broad focus on identifying, to the best of our ability, any policy actions related to COVID-19.

SPRC19’s second advantage comes from a focus on the policy process. It does this by coding actions–even if they do not change the policy in place. For example, even in the period covered, nearly 25% of actions are extensions. These do not change the current policy in place, but they offer potentially valuable insight into the dynamics of state policymaking. Information on the timing of policy implementation captures the evolution of states’ responses to the pandemic as well the different approaches taken by the states in developing and structuring their responses. This difference will be of particular interest for those who wish to examine how politics and other state features influence the policy choices state make. For example, some researchers may only be concerned with the presence or absence of a policy and not whether it occurred through an extension, but others studying the politics behind that policy may find the decision to extend to be critical. Policy solutions depend on within-state political preferences, conditions, and structures. They also reflect choices made by other states within the U.S. federal system through the process of policy diffusion^9,10^. SPRC19 offers an unprecedented opportunity to understand how policy choices spread across states in response to a sudden shock in which political leaders had very little information on which decisions to make.

The current public release of SPRC19 (v3.0) covers the period from January through April 2020. While it currently reflects a modest (albeit critical) time frame compared to other COVID-19 policy databases, it includes over 13,000 state policy actions drawn from nearly 4,000 documents. Moreover, our research team is actively working to extend the data for future updates.

## Methods

### Data collection

Our data collection began in March 2020. We aimed to capture every state policy action in response to COVID-19 that we could find, including executive orders, agency rules, new legislation, and court decisions. Our primary resources of data collection have been the websites (and their policy documents or press releases) of state political entities, namely governors, executive agencies, state legislatures, and state courts. We searched for official policy documents but also rely on press releases because of variation in resources across states. For some states, press releases are an important form of communication: smaller executive agencies, for instance, tend to share their updates with short news items rather than storing public documents. We prioritized locating the official policy text, but we used press releases as needed.

Specifically, we compiled a list of the websites for the various state actors from each state’s three branches of government. Most policies are drawn from executive orders from the Governor’s website, but many others are drawn from state judicial branch websites and state agency websites. The most frequently cited agencies are the Department of Public Health, the Department of Transportation, the Department of Motor Vehicles, State Parks, the Department of Corrections, the Department of Justice, and the Public Service Commission. After compiling these lists, we assigned research assistants to routinely check each of these websites and catalogue any documents found associated with new policies or policy updates. We established a systematic file-naming and categorization protocol for cataloguing files. While downloading policy documents, we also reviewed multi-state websites supporting state officials, such as the Council of State Governments, and cross-checked our updates from state websites with the National Conference for State Legislators’ State Action on Coronavirus along with several other policy specific resources like the Federation of State Medical Boards to help ensure we were not missing any updates or documents.

For each policy action identified, we download the source document–a bill, executive order, or press release–and then began coding it based on an evolving list of (in the current release) 206 distinct policies that we identified. The list of policies evolved as we downloaded and coded new documents. As research assistants coded documents, they kept in constant conversation through a Microsoft Teams channel about possible new policies to add and policies that did not fit current categories. If there was sufficient activity among states and if the policies seemed distinct enough, then we added a new policy category. We also revisited the current list of policies to decide whether some categories should be conslidated or removed due to infrequent use (typically removing those that were only used once, for example). In addition to coding each action by policy, we document the branch of government, the source document, the date of announcement, the implementation date, and an expiration date (if listed in the source document). We also note whether the policy action represents the introduction of a new policy or a change to an already-enacted policy. Those changes could involve an increase or decrease in the restrictiveness of the original policy, an extension of the current policy, or its repeal.

### Training

To prepare graduate and undergraduate students at the University of Iowa and the University of Notre Dame for these tasks, all new coders and supervisors went through a similar training process. This involved coding all sourcefiles collected for the state of Tennessee for the period ending April 29, 2020. These thirty four files had already been double coded by two experienced coders. The training set was eventually expanded as coding moved forward beyond the period included in the current release to include a total of sixty documents released through August 30, 2020 in order to encounter a greater variety of policies and action types. We chose Tennessee as our training set because its policy documentation has a straightforward structure to help coders can get used to the process of coding policies and policy actions. The training process took an average of about 20 hours. After coding each of the practice documents, new coders worked with a trained coder to refine and improve the accuracy their coding. This training made coders familiar with the policies, policy actions, and document formats before they were assigned to new states. This training also helped ensure coders were consistent (i.e., if two coders read the same block of text, they would code the same policy and action). To further ensure consistency, trained coders would periodically re-code documents that had already been coded by someone else. Communication through email, Microsoft Teams, and team meetings was used to facilitate answering common questions, incorporating new policy categories, and address any inconsistencies identified during re-coding.

### Data entry and coding

Data entry proceeded as follows. Coders were assigned a state and asked to code documents through a certain time period, initially April 2020 and later expanded to August 2020. They proceeded through documents chronologically for two reasons. First, to build familiarity with the style and formatting of that state’s documents. Second, and most importantly, to provide context on how to code sequences of actions on a given policy, i.e., whether the current action merely constituted a extension or whether it involved an increase or a decrease in a policy’s restrictiveness relative to the previous action. These practice documents covered this time period to ensure new coders were sufficiently trained on the different policy actions that became more prevalent at different time periods. For example, early COVID policies had many adoption actions while later policies were more likely to have or changed restrictiveness.

**Fig. 1:**
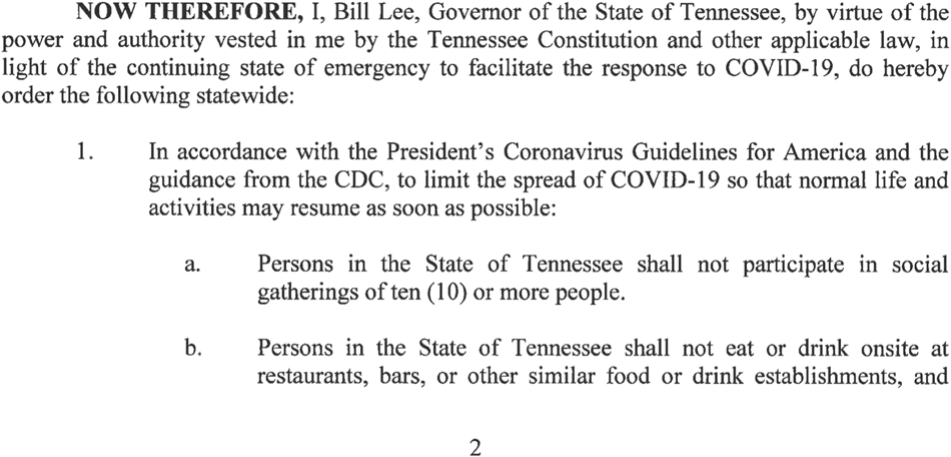
Sample Policy Text from Tennessee Executive Order 17 (2020).

For each published policy document, coders enter the document name and the source branch of government. Then, they read through each section or subsection to identify policy actions and code actions into the appropriate policy area. Figure 1, for example, is part of the 17th Executive Order by Tennessee Governor Bill Lee. In section 1-a, this order states not to socially gather with ten or more people. After reading this section, coders use our list of policies and identified the best fitting policy area. This case best fits the “gathering ban” policy category, which we define as “banning on gatherings of [size] and can also include policy related to state agencies’ authority to cancel gatherings that are in violation of these policies.” Defining our list of policies was an iterative process, wherein coders were instructed to flag sections that they thought warranted creation of a new policy, such as when a state issued a policy action that did not fit any of our already established categories. Our objective in establishing the list of policies was to define them narrowly enough to indicate a specific policy choice that could be compared across states, but not so narrowly such that each policy in a state could have no others like it.

After identifying an action for a specific policy area, coders next record the type of action: adoption, repeal, extension, increased restrictiveness, or decreased restrictiveness. If there is not yet a policy adoption for the given category for the given state, the policy action is coded as an *adoption*. If there is an adoption but no *repeal* yet recorded, coders compared the previous document and the current document to decide whether it imposes more restrictions (*increase restrictiveness*) or fewer restrictions (*decrease restrictiveness*) or whether it merely extends the previous policy’s expiration date (*extension*). With a gathering ban example, if the next order states no social gathering with more than five people, coders mark its policy action as an increase in restrictiveness because it imposes stronger restrictions than the previous policy. Alternatively, if the next document allows more than twenty people, it is coded as a decrease in restrictiveness. If the following policy document retains the current gathering ban level but extends the expiration date, it is coded as an extension. Finally, unlike many other widely used general policy adoption databases^9,11^, ours allows for repeated adoptions from a single state. For instance, there are occurrences where a state adopts, repeals, and then re-adopts a policy.

**Fig. 2:**
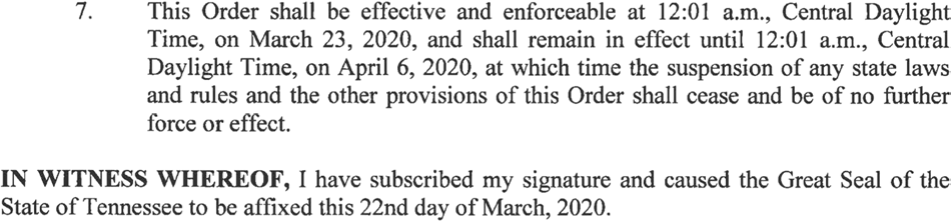
Example Policy Action Date Coding from Tennessee Executive Order 17 (2020).

SPRC19 also includes temporal information such as when the policy is signed or published (*announce_date*), when the policy becomes effective (*effective_date*), and when it expires (*expire_date*). Figure 2 shows an example of how executive order documents commonly conclude and how we code them. From section 7 of the excerpted order, coders mark “03-23-20” as the effective date and “04-06-20” as the expiration date. The announcement date is “03-22-20” based on when the document was published to the state website or based on the signature date on the document. While most policy documents have an announcement date, some do not provide clear effective and/or expiration dates. For the former, we assume the effective date is equivalent to the announcement date. For the latter, our coding reflects whether no expiration date is given or whether the expiration is expressed in terms of other conditions, such as when the state of emergency is ended. Specific text to this effect is included in our *expire_date_notes* variable.

After collecting and coding the data we incorporated additional information by grouping our policy areas into higher-level categories, drawing from other COVID-19 policy collections. We did so in two stages. Our intermediate level resulted in eighteen categories such as travel restrictions, prison policies, and medical licensing. We then grouped these into five high-level categories: containment and closure, economic, government/state operations, health system, and social.

It is important to highlight a key feature of our data collection and coding experience. The policy categories that we have identified and the higher-level categories we group them into have all emerged from the data and evolved as our data collection and entry have progressed. The pandemic and states’ responses to it are both on-going, evolving phenomena. Our categories were necessarily revised and expanded as coding stretched into 2021 to accommodate new types of policies. They were also regularly re-evaluated to determine whether policy areas could be consolidated or dropped. Policies enacted in April 2020 differ substantially from those debated in April 2022. Our data collection and coding evolved to reflect this fluidity. For example, many states began to enact bans on certain policies, such as local governments imposing curfews or limiting gatherings. As this became apparent as coding moved past the period included in the current release, we introduced new policy areas to represent the adoption of such bans, a few instances of which appear prior to April 2020.

## Data Records

Current and future releases of our data can be accessed via the SPRC19 Dataverse page^12^. The SPRC19 data are posted as a stand-alone data set within this Dataverse. It includes the policy actions data in Microsoft Excel and Stata formats with the option to export the latter in other formats. We also include our documentation file and a copies of the referenced sourcefiles, most of them in pdf format. The sourcefiles are stored within their own subfolder, *sourcefiles*, containing separate folders for each state, e.g., *sourcefiles/IA*. The folder structure is best viewed in the “Tree” view. The data and documentation files can be identified through by filtering for them in the “Table” view. The variables within the data file are as follows.

- **state**: State postal code.
- **statenam**: State name.
- **number**: Document identification code, which represents the state, release date, branch, and corresponding document number.
- **policy**: Policy area as coded from the full list of policies, which can be found in the codebook.
- **action**: The effect of the action on its assigned policy: adoption, an extension, repeal, increase in restrictiveness, or decrease in restrictiveness. Some documents may move a policy in multiple directions on the same day.
- **branch**: The branch of government issuing the policy: executive, judicial, or legislative.
- **agency**: If the document is written by an executive agency, this contains the related agency’s name (ex. Department of Health). This variable is left blank if the document is an executive order from the governor or if it is from a branch other than the executive.
- **announce_date**: Published date of the source document.
- **effective_date**: Effective date for the policy action.
- **expire_date**: Expiration date for the policy action. Expiration dates are not always specified in policy texts; we also left this entry blank if policies were specified to end with the lifting of the State of Emergency.
- **expire_date_range**: For when the expiration date includes a range or multiple possible values, e.g., Nevada Executive order from March 20, 2020 that tied the end of a school employee order to approval of school openings by the Chief Medical Office.
- **expire_date_notes**: Notes on expiration dates, e.g., “90 days after effective date” or “30 days after termination of state of emergency”.
- **retroactive**: Indicates if the policy goes into effect before the announcement date/filing of the policy (“yes” or blank).
- **sourcefile**: Source document file name including extension.
- **notes**: Additional notes on coding details and for users of the data.
- **policy_cat_med**: Policy category – Intermediate level.
- **policy_cat_hi**: Policy category – High level.

## Technical Validation

In order to check the accuracy of our data, we conducted a number of validation exercises, updating as needed, and then repeating them on subsequent iterations of the resource. Our first set of checks focused on ensuring that entered data all fit within the defined values and ranges. We did this in two ways. First, we used the validation tool in Excel to provide drop-down lists for policy areas, action types, and branches of government. This helps ensure uniformity of the data entered. It also provided coders easy access to the extensive list of policies to choose from. We also used the validation tool to evaluate policy announcement, expiration, and effective dates for out-of-range (i.e., prior to 2019 or after 2024) or otherwise unexpected values. In both cases unexpected entries produced a pop-up message prompting the coder to double check the entry or confirm that the value was appropriate. Our second validation was performed in Stata on the entered data after the fact. We compared the list of entered states, branches, actions, and policies to our definitive lists and corrected any errors. We checked whether the state code in the name of the source file matched the state name in the relevant worksheet. We checked all dates to confirm that they had appropriate values or ranges. We identified entries with unexpected missing values and corrected them. Once this cleaning was complete we also checked for duplicate entries to review and correct as needed.

For our second validation exercise, we checked our dates for gaps by identifying cases in which a state’s action on a given policy had an effective date that occurred more than a day after the previous action had expired. That is, if we have an entry extending a policy that expired two days ago, we checked whether that prior expiration date or the current effective date were correct. In many cases they were. For example, Iowa’s governor issued an order on May 13, 2020 that extended a closure order for casinos (see IA-051320-E-NO0001.pdf). That order listed an expiration date of May 27. On May 26, a new order allowed casinos to reopen at 50% capacity on June 1 effective through June 17 (see IA-052620-E-NO0001.pdf). Thus, there is a gap of five days between the expiration of the former order and the effective date of the new order. Iowa has over fifty such gaps in our existing data. In total, we found three hundred ninety-one such gaps that began during the period through April 30, 2020. This amount corresponds to just over three percent of all entries during the time period. We use data entered after April 30 to identify gaps because a sequence of policy actions may produce a gap only when a subsequent action occurs after April 30. We reviewed these gaps and found that twenty-three of them were appropriate, three hundred fifty-seven required corrections to our entry, and eleven required recodings of our entries.

Third, we compared our data to other published sources for policies that overlapped. Given the difference in how topic areas are created and what they include, combined with the much greater (and therefore often more nuanced) number of topic areas that we consider, these comparisons were limited to a small subset of topic areas. Our primary check involved the COVID AMP dataset. We identified over a dozen common topic areas that appeared to be similar based on their descriptions. From these we did a detailed comparison of state actions for four of them: curfews (ten), hazard pay (twelve), safer at home (seventy-six), and sick leave (sixty-seven). We examined all entries in both datasets through April 30, 2020 (one hundred sixty-five total) to identify differences based on announce dates, effective dates, end dates, and the source of the policy.

Our comparison revealed substantial differences between the two collections. We coded these by whether they resulted from differences in policy definitions, missing entries, or conflicting details. Differences in policy definitions explained the presence of entries regarding curfews in SPRC19 despite none in COVID AMP–but not in the other areas–so we set that one aside. The majority of differences between the two sources resulted from missing entries in one or the other. SPRC19 had the broader coverage, with the differences resulting from a wider range of policy declaration documents from which to identify actions, most notably the presence of many gubernatorial press releases declaring policy adoptions or extensions. These instances were especially common during the initial pandemic response. For cases with conflicts or missing entries in SPRC19, we reviewed the source documents and made appropriate corrections.

## Usage Notes

SPRC19 offers a highly detailed examination of the U.S. states’ response to the COVID-19 pandemic. Despite current data entry being complete only through April 2020, it already includes many times as many policy actions as most other datasets. This level of detail offers a fine-grained understanding of when and how the states enacted policies to address the pandemic. The broad policy coverage offers researchers a highly detailed picture of the actions states took. Many of these may have had smaller effects on virus transmission or deaths, but many will have had smaller effects in general or larger effects in more targeted areas. These and other details will also make SPRC19 valuable for researchers interested in the process and outcomes of governing during a pandemic^13,14^. For example, while many other datasets indicate when a particular intervention is in place, SPRC19 seeks to capture every extension, expansion, or contraction during that time period and which branch of government enacted it. These dynamics will be particularly useful for scholars interested in the process of the response. For example, why did some states make extensive use short-term orders with several extensions? And why did they often lead to short-terms gaps in policy orders? How did the branches of government work with or compete against each other as the pandemic response evolved? Many states experienced ongoing tension over authority to enact or repeal measures between the executive and legislative branches, with the courts sometimes brought in to arbitrate.

The scope of SPRC19 offers some clear advantages. First, it includes a range of policy actions that go beyond public-health-related NPIs typically included in extant data collections to include policies related to the consequences of the pandemic. Existing resources have wide variation in the number of policies they include, but SPRC19 has more than most, perhaps all of them. Moreover, the organization of the SPRC19 data facilitates analysis at the policy level; while some existing resources include information about most of the policies included in SPRC19, they often group them together into one policy area rather than devoting separate policies for each (e.g., COVID AMP^7^). SPRC19 therefore offers the opportunity for more granular information regarding state policy in specific topic areas, especially for researchers interested in those beyond the major interventions. Second, by doing so, SPRC19 allows researchers to study the political process in detail by describing the timing of actions for state policies in addition to their presence or absence, e.g., the timing of extensions. Such information on policy implementation captures the evolution of states’ responses to the pandemic as well the different approaches taken by the states in developing and structuring their responses. This difference will be of particular interest for those who wish to examine how politics and other state features influence the policy choices they made. The information contained in SPRC19 matters for understanding policymaking and governance. For example, some researchers may only be concerned with the presence or absence of a policy and not whether it occurred through an extension, but others studying the politics behind that policy may find the decision to extend to be critical.

This approach to documenting policy actions produces some features researchers using SPRC19 should be aware of. Most notably, those interested in studying the timing of state actions on some or all of the included policies should be aware that states may take multiple actions on the same policy on the same day. These may come from separate branches or government, but on rare occasions they may even come from the same branch. In some cases, a single document may even specify multiple actions on the same policy.

In contrast, those interested in some details about the U.S. response to COVID-19 may prefer other resources. SPRC19 focuses on state policy responses and therefore includes no information on Federal or local responses. It also does not systematically record the exact level or target population for a given policy. For example, some resources report the precise maximum gathering size or specific age ranges to which a given policy applies. Such information may be critical for addressing some research questions, but is not recorded in SPRC19.

## Data Availability

No custom code was used. Software tools used for processing are mentioned in the Methods and Technical Validation sections.

## References

[1] Zheng Q (2020). HIT-COVID, a global database tracking public health interventions to COVID-19. Scientific Data.

[2] Desvars-Larrive A (2020). A structured open dataset of government interventions in response to COVID-19. Scientific Data.

[3] Cheng C, Barceló J, Hartnett AS, Kubinec R, Messerschmidt L (2020). COVID-19 government response event dataset (CoronaNet v.1.0). Nature Human Behaviour.

[4] Raifman J (2021). ICPSR.

[5] Skinner A (2022). A database of US state policies to mitigate COVID-19 and its economic consequences. BMC Public Health.

[6] Hale T (2021). A global panel database of pandemic policies (Oxford COVID-19 Government Response Tracker). Nature Human Behaviour.

[7] The Georgetown University Center for Global Health Science and Security, Talus Analytics, The Nuclear Threat Initiative & COVID Act Now. The COVID analysis and mapping of policies (2021).

[8] Fullman, N. *et al*. State-level social distancing policies in response to COVID-19 in the US (2021).

[9] Walker JL (1969). The diffusion of innovations among the American states. American Political Science Review.

[10] Desmarais BA, Harden JJ, Boehmke FJ (2015). Persistent policy pathways: Inferring diffusion networks in the american states. American Political Science Review.

[11] Boehmke FJ (2020). SPID: A new database for inferring public policy innovativeness and diffusion networks. Policy Studies Journal.

[12] Boehmke FJ (2022). Harvard Dataverse.

[13] Adolph C, Amano K, Bang-Jensen B, Fullman N, Wilkerson J (2021). Pandemic politics: Timing state-level social distancing responses to COVID-19. Journal of Health Politics, Policy and Law.

[14] Patterson S (2022). The politics of pandemics: The effect of stay-at-home orders on COVID-19 mitigation. State Politics & Policy Quarterly.

